# A Microtube Array Membrane (MTAM) Encapsulated Live Fermenting *Staphylococcus epidermidis* as a Skin Probiotic Patch against *Cutibacterium acnes*

**DOI:** 10.3390/ijms20010014

**Published:** 2018-12-20

**Authors:** Albert-Jackson Yang, Shinta Marito, John-Jackson Yang, Sunita Keshari, Chee-Ho Chew, Chien-Chung Chen, Chun-Ming Huang

**Affiliations:** 1 Department of Biomedical Sciences and Engineering, National Central University, Taoyuan 32001, Taiwan; albert_depok@yahoo.co.id (A.-J.Y.); shintasimbolon53@yahoo.com (S.M.); 2 Department of Life Sciences, National Central University, Taoyuan 32001, Taiwan; johnjacksonyang@gmail.com (J.-J.Y.); sunitakeshari827@gmail.com (S.K.); 3 Graduate Institute of Biomedical Materials &Tissue Engineering, Taipei Medical University, Taipei 11031, Taiwan; chchew88@gmail.com; 4 Department of Dermatology, School of Medicine, University of California, San Diego, CA 92121, USA

**Keywords:** acne, fermentation, *C. acnes*, probiotic, PSF MTAM, *S. epidermidis*

## Abstract

Antibiotics without selectivity for acne treatment may destroy the beneficial microbes in the human microbiome that helps to fight *Cutibacterium acnes* (*C. acnes*), a bacterium associated with inflammatory acne vulgaris. Probiotic treatment by direct application of live *Staphylococcus epidermidis* (*S. epidermidis*) onto the open acne lesions may run the risk of bloodstream infections. Here, we fabricated the polysulfone microtube array membranes (PSF MTAM) to encapsulate probiotic *S.* *epidermidis.* We demonstrate that the application of the encapsulation of *S. epidermidis* in PSF MTAM enhanced the glycerol fermentation activities of *S. epidermidis.* To mimic the granulomatous type of acne inflammatory acne vulgaris, the ears of mice were injected intradermally with *C. acnes* to induce the secretion of macrophage inflammatory protein-2 (MIP-2), a murine counterpart of human interleukin (IL)-8. The *C. acnes*-injected mouse ears were covered with a PST MTAM encapsulated with or without *S.* *epidermidis* in the presence of glycerol. The application of *S.* *epidermidis*-encapsulated PST MTAM plus glycerol onto the *C. acnes*-injected mouse ears considerably reduced the growth of *C. acnes* and the production of MIP-2. Furthermore, no *S. epidermidis* leaked from PSF MTAM into mouse skin. The *S. epidermidis*-encapsulated PST MTAM functions as a probiotic acne patch.

## 1. Introduction

The skin microbiota is a variety of microorganisms in the skin ecosystem. Many of these microorganisms are harmless and in some conditions provides crucial functions that the human genome has not evolved. Some symbiotic microorganisms such as fermenting bacteria confer protection against invasion of pathogenic microorganisms [1]. Partly, the skin condition that is influenced by various internal and external factors controlled the stability of the skin microbiome, in the other is synergistic and antagonistic interactions of host with the microbiota. Inhibitory substances, including bacteriocins that are produced by skin microorganisms may help to prevent colonization by pathogens [2]. The inflammatory acne vulgaris that links to the over-growth of *C. acnes* (formally named as *Propionibacterium acnes*) affects an estimated 80% of Americans [3]. Excess sebum production, follicular hyperkeratinization, and androgenic stimulation also play a role in the pathogenesis of acne vulgaris [2]. 

When considering the involvement of *C. acnes* in the etiology of acne vulgaris [4], antibiotics have always been used as an integral part of the therapy. Topical antibiotics are prescribed for acne vulgaris with mild inflammatory lesions, while systemic or oral antibiotics, such as doxycycline, minocycline, azithromycin, cephalosporins, and fluoroquinolones are used for moderate to severe disease [5]. Other treatments include antibiotics with benzoyl peroxide or azelaic acid for the reduction of development of resistant strains [4] and oral corticosteroids for severe inflammatory acne vulgaris [6]. The isotretinoin, a retinoid with known side-effects, has been commonly used for the treatment and prevention of severe acne [7]. Recently, probiotics have been used as an alternative treatment for acne vulgaris to reduce the risk of developing antibiotic-resistant *C. acnes*. Results in previous studies demonstrated that commensal microorganisms in the human skin microbiome could mediate the fermentation of glycerol that is naturally produced in the skin to exert the inhibitory effects on the over-growth of *C. acnes*. *Staphylococcus epidermidis* (*S. epidermidis*), a skin probiotic bacterium in the human skin microbiota, can ferment glycerol and create inhibition zones to repel *C. acnes*. Succinic acid, one of four short-chain fatty acids (SCFAs) detected in media of *S. epidermidis* glycerol fermentation effectively inhibits the growth of *C. acnes* in vitro and in vivo [8,9].

As mentioned above, antagonism interaction between *S. epidermidis* and *C. acnes* has significance interferences for the growth of *C. acnes*. According to Christensen et al., the majority of *S. epidermidis* strains were able to inhibit *C. acnes* [10]. The result is in agreement with that reported by Rajiv et al., in that *S. epidermidis* is a part of the human skin microflora that has an inhibitory effect on the growth of *C*. *acnes* [11]. Wang et al. showed that the level of macrophage-inflammatory protein-2 (MIP-2) and the number of *C*. *acnes* in mouse ears that were injected with *S*. *epidermidis* and *C*. *acnes* in the presence of sucrose were considerably lower than those in ears injected with this two bacteria plus phosphate buffered saline (PBS) [12]. However, live *S*. *epidermidis* as a probiotic, when added exogenously into the open lesion/comedone of acne vulgaris, might not be a viable option in patients with significant underlying health issues, such as surgical intervention and immune suppression. Furthermore, the Food and Drug Administration (FDA) has not yet approved the live probiotic bacteria for topical therapeutic use. Although killed probiotic bacteria have been topically applied onto skin for stimulation of the immune cells and eradication of colonized pathogens [13], the bacterial lysates may not able to directly hinder the growth of invading pathogens. 

To circumvent the problems above, we use the new class of hollow fiber, known as microtube array membrane (MTAM), which can offer the potential to immobilize and capsulate living microorganism. In this study, MTAM is utilized to encapsulate the *S*. *epidermidis*, allowing for bacterial fermentation to occur in the MTAM and achieving the sustained release of fermentation metabolites from MTAM. MTAMs with similar but superior to hollow fibers have been developed using coaxial electrospinning [14,15]. They are made of hundreds of highly aligned, nano-porous, ultra-thin, one-to-one connected fibers that are self-assembled in a single layer. Unlike a typical hollow fiber, MTAMs are one to two orders smaller in diameter and greater than two orders thinner in the tube wall [16,17]. They have been further functionalized by generating nanopores on the surface and are followed by a leaching out process. The poly-l-lactic acid (PLLA)-based MTAM has been used to immobilized yeasts [18]. This was a novel and promising technology for bioethanol fermentation in vitro. 

In this study, we used polysulfone (PSF) as material to assemble the polysulfone microtube array membranes (PSF MTAMs). PSF has been commonly used in bio-industry for ultrafiltration and bacterial microencapsulation, allowing for two directional diffusions of molecules dynamically. Highly aligned property and highly specific surface area of PSF MTAM made it a suitable microbe carrier. Here, we take advantage of PSF MTAM to encapsulate *S*. *epidermidis* and create it as a skin probiotic patch. We envision that *S*. *epidermidis* will be trapped within PSF-MTAM without directly contacting the skin, while the PSF-MTAM-based skin patch is topically applied onto the skin.

## 2. Results

### 2.1. The S. epidermidis-encapsulated PSF MTAM

**Figure 1:**
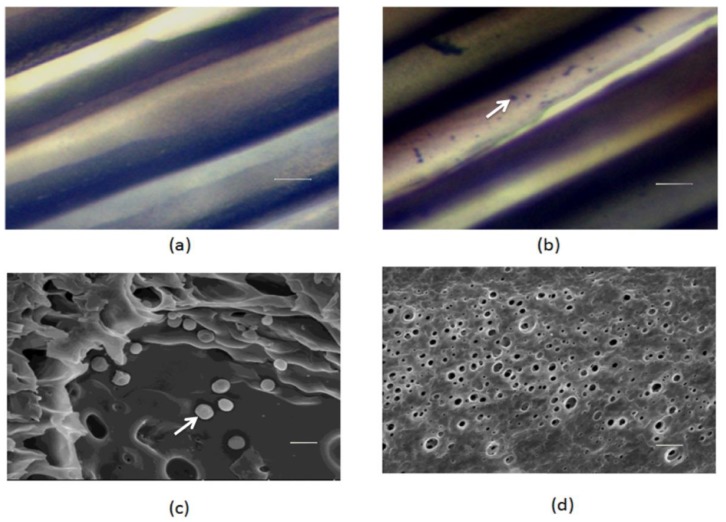
The light microscopy images of *S. epidermidis* (**a**) un-loaded and (**b**) loaded polysulfone microtube array membranes (PSF MTAMs) and the scanning electron micrographs (SEM) of inner (**c**) and outer (**d**) surfaces of *S. epidermidis*-loaded PSF MTAMs. Arrows indicate the *S. epidermidis*. Bars (**a**,**b**) = 10 µm; and (**c**,**d**) = 1 µm.

The lumen surfaces of PSF MTAM with or without *S. epidermidis* encapsulation (Figure 1a) were stained by a Gram staining kit and visualized using optical microscopy. *S*. *epidermidis*, a gram positive bacterium, which is immobilized on the surface of PSF MTAM lumen, was stained by the crystal violet (Figure 1b). The scanning electron microscope (SEM) was utilized to image the nano-porous structure of the outer surface of PSF MTAM (Figure 1d) and confirm the immobilization of *S. epidermidis* on the inner surface of PSF MTAM (Figure 1c). 

### 2.2. Glycerol Fermentation of S. epidermidis in PSF MTAM

**Figure 2:**
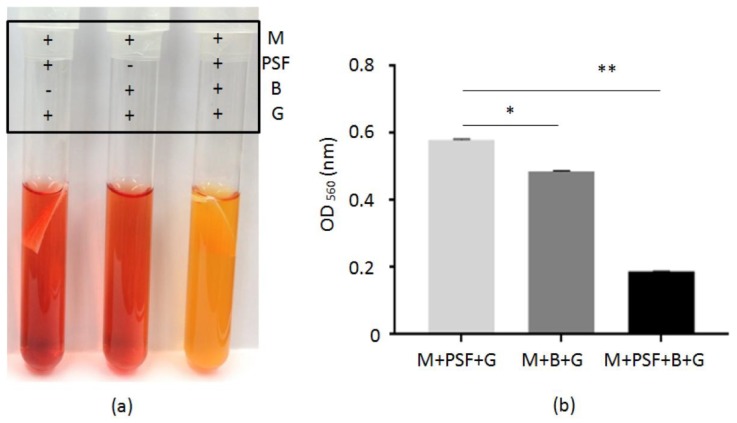
Fermentation activity of *S. epidermidis*. (**a**) PSF MTAM loaded-bacteria (B) (10^5^ colony-forming unit (CFU)/mL) were incubated in phenol red-containing rich media (M) with glycerol (G). Rich media plus glycerol with a bacteria-unloaded PSF MTAM (PSF) alone or bacteria alone were included as controls. *S. epidermidis* fermentation on 10 h was displayed. A yellow color change in media is indicative of extensive fermentation of bacteria. (**b**) The OD_560_ value in the media with glycerol plus PSF MTAM loaded-bacteria (M+PSF+B+G) was significantly lower than those in the media with glycerol plus PSF MTAM (M+PSF+G) or bacteria (M+B+G). Results were illustrated as the mean ± standard derivation (SD) of three independent experiments. * *p* < 0.01; ** *p* < 0.001 (two-tailed *t*-tests).

To examine whether when *S*. *epidermidis* (ATCC 12228) bacteria were encapsulated in PSF MTAM, they still retain their fermentation activities. Bacteria encapsulated in PSF MTAM were incubated in the presence of 2% glycerol in media containing phenol red. Controls include media with PSF MTAM alone or suspended bacteria alone. In agreement with previous results [8], the suspended *S*. *epidermidis* can use the glycerol as a carbon source for fermentation, as indicated by the color change of phenol red in media from red to orange after 10 h culture. No color change occurs in the media with PSF MTAM alone. More significantly, the media with bacteria encapsulated in PSF MTAM turns yellow 10 h after culture, suggesting that fermentation becomes more efficient when *S. epidermidis* were encapsulated in PSF MTAM. The color change of phenol red was quantified by optical density (OD)_560_. As shown in Figure 2b, a greater decrease in the value of OD_560_ was detected when *S*. *epidermidis* were encapsulated in PSF MTAM in the presence of glycerol, indicating the bacteria immortalized on the internal surface of PSF MTAM perform more effective fermentation. 

### 2.3. In Vivo Inhibition of C. acnes Growth and Inflammation by S. epidermidis Fermentation 

**Figure 3:**
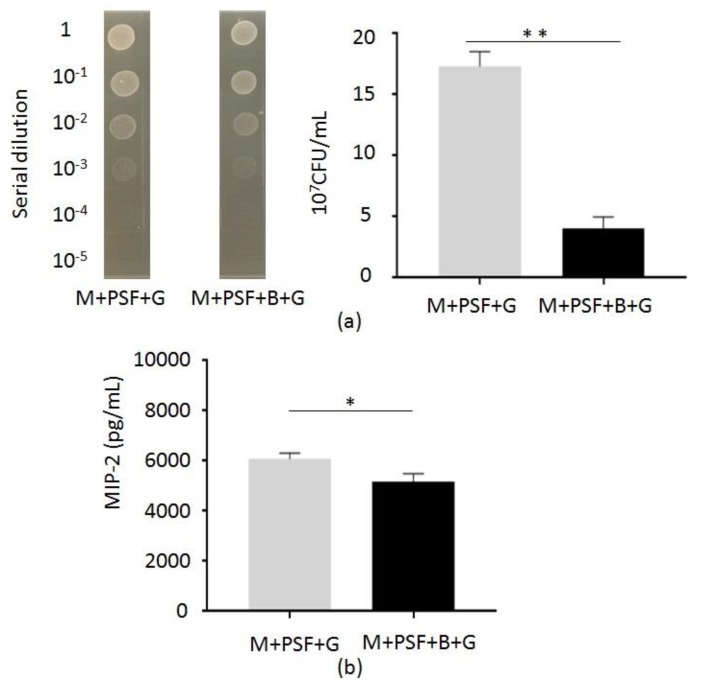
Reduction of *C. acnes* colonization and inflammation in vivo by glycerol fermentation of *S. epidermidis* in PSF MTAM. The ears of Institute for Cancer Research (ICR) mice were injected intradermally with *C. acnes* (ATCC 6919) (10^7^ CFU) and topically applied with PSF MTAM loaded with (M+PSF+B+G) or without (M+PSF+G) *S. epidermidis* (ATCC 12228) (10^5^ CFU) in the presence of 2% glycerol in rich media. Three days after *C. acnes* injection, (**a**) The CFUs were enumerated by plating serial dilutions (1:10–1:10^5^) of the ear homogenate on an agar plate. (**b**) The levels of MIP-2 cytokines in the ears were measured by an enzyme-linked immunosorbent assay (ELISA) kit. Data are the means of three separate experiments using three mice per group. * *p* < 0.01; ** *p* < 0.001 (two-tailed *t*-tests).

To examine the *S. epidermidis* fermentation against *C*. *acnes* in vivo, the ear of Institute for Cancer Research (ICR) mice was injected intradermally with *C*. *acnes* (ATCC 6919) (10^7^ CFU) and applied with a PSF MTAM that was encapsulated with or without *S. epidermidis* (ATCC 12228) (10^5^ CFU) in the presence of 2% glycerol in rich media. Three days after *C. acnes* injection, ears were excised and homogenized for the quantification of the number of *C. acnes* and the level of macrophage-inflammatory protein-2 (MIP-2), which is a murine counterpart of human interleukin (IL)-8. As shown in Figure 3a, the number ((4.1 ±1.5) × 10^7^ CFU) of *C. acnes* in mouse ear applied with *S. epidermidis*-encapsulated PSF MTAM was lower than that ((17.3 ± 2.1) ×10^7^ CFU) in ear applied PSF MTAM un-loaded with *S. epidermidis*, demonstrating the in vivo activity of a *S. epidermidis*-encapsulated PSF MTAM as a skin probiotic patch against *C. acnes*. 

To determine whether the application of *S. epidermidis*-encapsulated PSF MTAM in the presence of glycerol can regulate the *C*. *acnes*-induced inflammation, mouse ears were excised and homogenized three days after application for measurement of the level of MIP-2 by ELISA. The amount ((5.18 ± 0.39) × 10^3^ pg/mL) of MIP-2 in the ear applied with *S. epidermidis*-encapsulated PSF MTAM 14.8% less than that ((6.08 ± 0.54) × 10^3^ pg/mL) in the ear applied with a PSF MTAM un-loaded with *S. epidermidis* (Figure 3b).

### 2.4. Detection of Bacterial Leaking from PSF MTAM 

**Figure 4:**
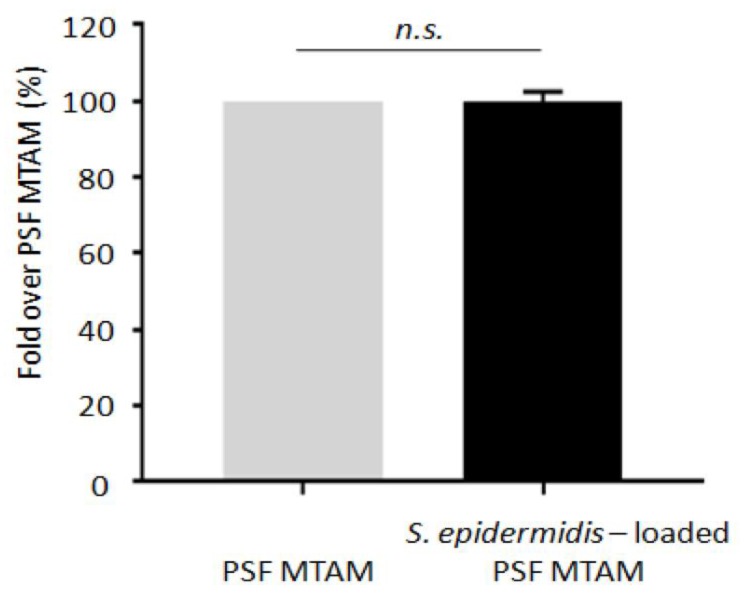
No bacterial leaking from *S. epidermidis*-loaded PSF MTAM. The ears of ICR mice were topically applied with PSF MTAM or *S. epidermidis*-loaded PSF MTAM. Three days after application, the number of *S. epidermidis* in ears was quantified by qRT-PCR. n.s. = non significant. (two-tailed *t*-tests).

To prove that no *S. epidermidis* was leaking from PSF MTAM to skin, the ear of ICR mice was applied with a PSF MTAM that was encapsulated with or without *S. epidermidis* (ATCC 12228) (10^5^ CFU) in the presence of 2% glycerol for three days. DNA was extracted for real-time quantitative reverse transcription polymerase chain (PCR) reaction (qRT-PCR) using *S*. *epidermidis* specific primers. As shown in Figure 4, no significant difference in the number of *S. epidermidis* in ears that were applied with a PSF MTAM encapsulated with or without *S. epidermidis*, indicating that no encapsulated *S. epidermidis* bacteria leaked from PSF MTAM into mouse ears. 

## 3. Discussion

The bacterial interference in which beneficial bacteria are known are widely used for prevention or treats from infections pathogens [19,20,21,22,23,24]. Although probiotic bacteria have documented skin benefits, live bacteria are generally not preferred in cosmetics and are currently restricted by FDA. Rather than including live bacteria, many of the probiotic skin care formula use fermentation metabolites and less products using live bacteria directly for the therapy of skin problem [25,26]. Here, we demonstrate PSF MTAM as option to immobilize and capsulate live probiotic *S. epidermidis* for the suppression of growth of *C*. *acnes* and inflammation in mice. This technique may allow for the distribution of fermentation products from PSF MTAM to skin, while glycerol, a carbon source, is fermentatively metabolized to SCFAs. Examination of *S. epidermidis*-loaded and unloaded MTAM by Gram staining showed that the *S. epidermidis* was encapsulated inside the PSF MTAM (Figure 1a,b). The technique of bacterial immobilization using PSF MTAM has been used for fermentation acceleration. PSF MTAM was used to localize bacteria in a certain space and to maintain their catalytic activities. Immobilization of bacteria can increase bacterial density, shorten fermentation times, and increase fermentation products [18,27,28]. A previous publication from our laboratory has showed that *S*. *epidermidis* fermentation could effectively inhibit the growth of *C*. *acnes* in vitro and in vivo [8]. In this study, we observed the fermentation of *S*. *epidermidis* in media or PSF MTAM in the presence of 2% glycerol and phenol red. As shown in Figure 2, the fermentation of *S*. *epidermidis* encapsulated in PSF MTAM is more efficient than that of *S*. *epidermidis* in media without PSF MTAM encapsulation, demonstrating that PSF MTAM provides a microenvironment for the facilitation of fermentation of *S*. *epidermidis* (Figure S3). This result agrees with the previous publication that the fermentation of microorganisms can be accelerated when microorganisms were immobilized on the biomaterial surface [18,29,30]. Treatments of severe acne vulgaris by intralesional corticosteroid injection [31,32] can cause adverse effects. Although *S. epidermidis-*encapsulated PSF MTAM may not cover large areas of the skin with multiple acne lesions, the PSF MTAM can be applied on severe acne, like cystic acne, or an acne spot that is just starting to form. The *S. epidermidis-*encapsulated PSF MTAM can be also potentially used as an adjuvant that can be applied onto the acne lesions right after treatments of anti-acne drugs, such as salicylic acid, promoting drug efficacy. Glycerol can be naturally produced by skin cells [33]. The 0.7 and 0.2 μg cm^−^^2^ of glycerol can be detected on the surface of human cheek and forearm, respectively [34]. Although 2% glycerol was used in this study, future works will determine which concentration of glycerol is the most suitable dose to induce the *S. epidermidis* fermentation in PSF MTAM.

The anti-*C*. *acnes* activities of the majority of *S. epidermidis* strains have been previously investigated [10]. Publications from our laboratory demonstrated that *S*. *epidermidis* could ferment glycerol to SCFAs, including acetic, butyric, lactic, and succinic acids [8]. The SCFAs produced by *S*. *epidermidis* can attenuate *C*. *acnes*-induced inflammation. It has been reported that SCFAs can inhibit the histone deacetylase (HDAC) or activate of free fatty acid receptors (Ffar1; also known as G-protein coupled receptor 41 (GPR41) and Ffar2; GPR43) [12]. Succinic acid is an SCFA that is detected in media of *S*. *epidermidis* glycerol fermentation by nuclear magnetic resonance (NMR) analysis. Literature has shown that succinic acid significantly lowered the intracellular pH of *C*. *acnes*, supporting the conclusion that lowered intracellular pH of microbe is a lethal mechanism of SCFAs [8,35,36]. Topical application of succinic acid markedly suppressed the *C*. *acnes*-induced inflammation in mice. In this study, we injected *C*. *acnes* intradermally into mouse ears to induce the granulomatous type of inflammation [37,38,39]. The Elizabethan collars were used to prevent mice from accessing the PSF MTAM [40]. As shown in Figure 3b, application of *S*. *epidermidis* encapsulated PSF MTAM on mouse ear siginificanly dimisihed the *C*. *acnes*-induced MIP-2 production, suggesting that SCFAs that were released from PSF MTAM to skin attenuated the *C*. *acnes*-induced inflammation [41]. Results in our recent publication demonstrated that the levels of IL-8, a human counterpart of MIP-2, in acne lesional skin were noticeably higher than those in non-lesional skin in acne patients and normal skin in healthy subjects [42]. Since acne vulgaris is a skin inflammatory disease, the application of *S*. *epidermidis* encapsulated PSF MTAM for the reduction of *C*. *acnes*-induced inflammation illustrates the value of *S*. *epidermidis* encapsulated PSF MTAM as a patch for the treatment of inflammatory acne vulgaris. SCFAs were produced by skin cells and commensal bacteria in relatively low concentrations [43]. SCFAs in the human bloodstream have been detected at low level, ranging from 3 to 7 mM, and at higher level (20–140 mM) locally produced by the microbes in the colon [44]. A higher dose of SCFAs may be required to achieve in vivo efficacy due to its rapid metabolism by host cells [45,46]. It has been documented that SCFAs with short half-lives have apparent difficulty achieving pharmacologic concentrations in vivo [47]. Furthermore, it can be a challenge to formulate a mixture of SCFAs at the different concentration ratios when SCFAs can be developed as anti-*C*. *acnes* agents. 

Both PSF and poly-L-lactic acid (PLLA) can be used to fabricate MTAM [48]. When compared to PLLA, PSF has higher flexural, flexural, and tensile strength, making PSF easier to handle and modified for development of MTAM [49,50,51]. The brittleness of PLLA MTAM restrains its practical application, especially for a porous membrane with thin thickness and high porosity [52]. By contrast, PSF is one of polymers with hydrolytic resistance and dimensional stability [53]. To develop a skin probiotic patch for the treatment of acne vulgaris in the future, PSF MTAM was used a biomaterial for encapsulation of probiotic *S*. *epidermidis*. Encapsulation of *S*. *epidermidis* in PSF MTAM enhances the fermentation activities of bacteria and it prevents the direct contact of bacteria to skin.

## 4. Materials and Methods 

### 4.1. Culture of Microorganisms

*S*. *epidermidis* (ATCC 12228) was grown on tryptic soy broth (TSB) (Sigma, St. Louis, MO, USA) overnight at 37 °C *C*. *acnes* (ATCC 6919) was cultured in Reinforced Clostridium Media (RCM, Oxford, Hampshire, England) under an anaerobic condition using a Gas-Pak (BD, Sparks, MD, USA) at 37 °C. Bacterial pellets were harvested by centrifugation at 5000× *g* for 10 min, washed with PBS, and then suspended in PBS or TSB.

### 4.2. Fermentation of Bacteria

*S. epidermidis* (10^5^ CFU/mL) was loaded by the siphon technique into PSF MTAM, as previously described [54]. *S*. *epidermidis* in PSF MTAM was incubated in 10 mL rich media (10 g/L yeast extract (Biokar Diagnostics, Beauvais, France), 3 g/L TSB, 2.5 g/L K_2_HPO_4_, and 1.5 g/L KH_2_PO_4_), 2% glycerol, and the 0.002% (*w/v*) phenol red (Sigma) as a fermentation indicator. *S*. *epidermidis* alone or PSF MTAM alone without *S. epidermidis* in the presence or absence (Figure S2) of 2% glycerol in phenol red-containing rich media was included as a control. A color change from red-orange to yellow indicated the occurrence of bacterial fermentation and it was detected by OD_560_. 

### 4.3. Fermentation of S. epidermidis in PSF MTAM Against C. acnes In Vivo

The mouse work was carried out in strict accordance with an approved Institutional Animal Care and Use Committee (IACUC) protocol (NCU-106-015, 19 December 2017) at National Central University, Taiwan. ICR mice (8–12 month-old females; National Laboratory Animal Center, Taiwan) were anesthetized by isoflurane (Sigma). Three mice per group were used in each experiment. The ears of ICR mice were injected intradermally with *C. acnes* (10^7^ CFU) and were topically applied with *S. epidermidis*-encapsulated PSF MTAM (Figure S4). Topical application of a *S. epidermidis*-unloaded PSF MTAM on the other ear served as a control. An Elizabethan collar used to avoid a case that mice scratch out the PSF MTAM. Rich media with 2% glycerol was dropped onto PSF MTAM every 12 h. PSF MTAM was renewed every day for three days. Ears were excised, weighed, and homogenized for cytokine detection and/or bacterial counts. The total protein concentration was measured by a Pierce BCA Protein Assay Kit (Thermo Scientific, Waltham, MA, USA). 

### 4.4. SEM

The micro-structures of the PSF MTAM that were loaded with or without *S. epidermidis* were revealed using a SEM (SU8020, Hitachi, Tokyo Japan) at an accelerating voltage of 5 kV. The individual fiber diameter and pore size were measured on SEM images using Image J (National Institutes of Health, Bethesda, MD, USA).

### 4.5. Bacterial Loads in Mouse Ears

After excising mouse ears, tissue homogenates were made by a tissue grinder in 200 µL of sterile PBS. CFUs of *C. acnes* in ear homogenates were enumerated by plating serial dilutions (1:10–1:10^5^) of homogenates on a RCM plate. After that, plates were incubated for three days at 37 °C under an anaerobic condition using a Gas-Pak. Only *C. acnes* that were injected into mouse ear, but not other bacteria in PBS-injected mouse ear, can form colonies in RCM plates (Figure S1). A biosafety level 2 (BSL-2) facility was used for the conduction of mouse experiments in accordance with institutional guidelines for animal experiments.

### 4.6. ELISA

The pro-inflammatory MIP-2 cytokine was determined by sandwich ELISA using a Quantikine mouse MIP-2 set (R&D Systems, Minneapolis, MN, USA).

### 4.7. Detection of S. epidermidis Leaking from PSF MTAMs 

The ICR mice (three mice per group) were anesthetized by isoflurane (Sigma). Ears were topically applied with a *S. epidermidis* loaded- or unloaded-PSF MTAM. The experiment was conducted with mice wearing an Elizabethan collar. TBS with 2% glycerol was applied onto PSF MTAM every 12 h. PSF MTAM was replaced every day. After three days, the ears were excised, weighed, and homogenized for DNA extraction using an EasyPure Genomic DNA spin Kit (Bioman, Taipei, Taiwan). In each realtime PCR run, two independent samples were analyzed, and each experiment was performed twice independently. The primers sequences used for qRT-PCR were SepdivFW3 5’-TTCCGCTCTCGTTTCCGT-3’ and RTSepdivREV 5’-ATTGCACGTTCTTCAGGTGT-3’ [55]. The qRT-PCR was performed on StepOne^TM^ system (Applied Biosystems, Foster City, CA, USA) using a Power SYBR™ Green PCR Master Mix (Roche, Castle Hill, Australia). Briefly, 25 µL reactions contained 12.5 µL Master Mix, 10.5 µL RNase-Free Water, 0.5 µL, 0.75 µM forward primer and reverse primer and 1µL DNA sample. The qRT-PCR program consisted of an initial denaturation step at 95 °C for 10 min, followed by 40 cycles of amplification, and quantification at 95 °C for 15 s, 48 °C for 15 s, and 95 °C for 10 s. At the end of the program, a melt curve analysis was conducted. At the end of each qRT-PCR run, the data were automatically analyzed by the system, and an amplification plot was generated for each DNA sample. Threshold cycle or C_t_ values (StepOne^TM^ system) is defined as the number of cycles required for the fluorescent signal to cross the threshold (i.e., exceeds background level). C_t_ levels are inversely proportional to the amount of target nucleic acid in the sample. The Ct value was analyzed by StepOne^TM^ system. The fold expression or repression of the target gene relative to the control gene in each sample was calculated by the “fold-over-control” method [56].

### 4.8. Statistics

Experiments were repeated at least three times with similar results. Statistical significance was determined using Student’s unpaired two-tailed t-test, as indicated in the legend (* *p* < 0.01, ** *p* < 0.001).

## Supplementary Materials

Supplementary materials can be found at http://www.mdpi.com/1422-0067/20/1/14/s1.
