# Supplementary material for: A Microtube Array Membrane (MTAM) Encapsulated Live Fermenting Staphylococcus epidermidis as a Skin Probiotic Patch against Cutibacterium acnes
Source: Int J Mol Sci. 2018 Dec 20;20(1):14. doi: 10.3390/ijms20010014 (PMC6337527; doi:10.3390/ijms20010014)
Supplement: Supplementary file 1 [file ijms-20-00014-s001.pdf]

1. Supplementary Information

1.1. Detection of Bacteria in C. acnes-injected Mouse Ears

To confirm the bacterial colonies on RCM plates are *C. acnes*, the ears of ICR mice were injected intradermally with *C. acnes* (10^7 CFU) or PBS for three days. Injection of *C. acnes* induced redness (Figure 1Sa). Serial dilutions of ear homogenates were placed on RCM plates. No bacterial colonies grew when homogenates of ears injected with PBS were placed on RCM plates. On the other hand, $5.3 \pm 0.9 \times 10^7$ CFUs of bacterial colonies were detected when homogenates of ears injected with *C. acnes* were placed on plates (Figure 1Sb, c). The result indicates that commensal bacteria in mice may be not able to grow on RCM bacteria after isolation based on a protocol as described in Section 4.5, under Materials and Methods. The result also suggests that colonies form on RCM plates are *C. acnes* bacteria.

1.2. Glycerol as A Carbon Source for Fermentation of S. epidermidis

As shown in Figure 2, we found that *S. epidermidis* fermentation was enhanced when bacteria were encapulated in PSF MTAM in the presence of glycerol. Besides glycerol, other ingredients such as amino acids in media may be used by bacteria as carbon sources for fermentation. To examine whether glycerol is a mjaor carbon source from *S. epidermidis* fermentation, the *S. epidermidis* suspended in meda or encapulated in PSF MTAM were cultured in the presence or absence of 2% glycerol. The color change of phenol red due to a decrease in pH values was quantified by OD_{560}. In the absence of glycerol, bacterial growth in PSF MTAM for 3 days led to a drop in the value of OD_{560}. However, a significantly greater decrease in the value of OD_{560} was observed when either suspended or PSF MTAM-encapsulated *S. epidermidis* bacteria were cultured in the presence of glycerol (Figure 2S), indicating the glycerol is a major carbon source for fermentation of *S. epidermidis*.

1.3. The Growth of S. epidermidis in PSF MTAM

To determine if *S. epidermidis* can grow in PSF MTAM, the number of bacteria encapsulated in PSF MTAM was counted 10 (Figure S3a, b) or 20 (Figure S3c, d) h after incubation. The PSF MTAM encapsulated by *S. epidermidis* was homogenized for bacterial counts. The $4.7 \pm 0.6 \times 10^7$ and $2.3 \pm 0.6 \times 10^{10}$ CFU/ml of bacteria were detected after 10 and 20 h incubation, respectively, demonstrating that *S. epidermidis* survived and replicated in PSF MTAM. As shown in Figure S3, no remarkable difference in the number of *S. epidermidis* that were suspended in media or encapsulated in PSF MTAM for 10 or 20 h. The data suggested that PSF MATAM-induced enhancement of glycerol fermentation of *S. epidermidis* (Figure 2) is not because *S. epidermidis* grew faster in PSF MTAM than in media.

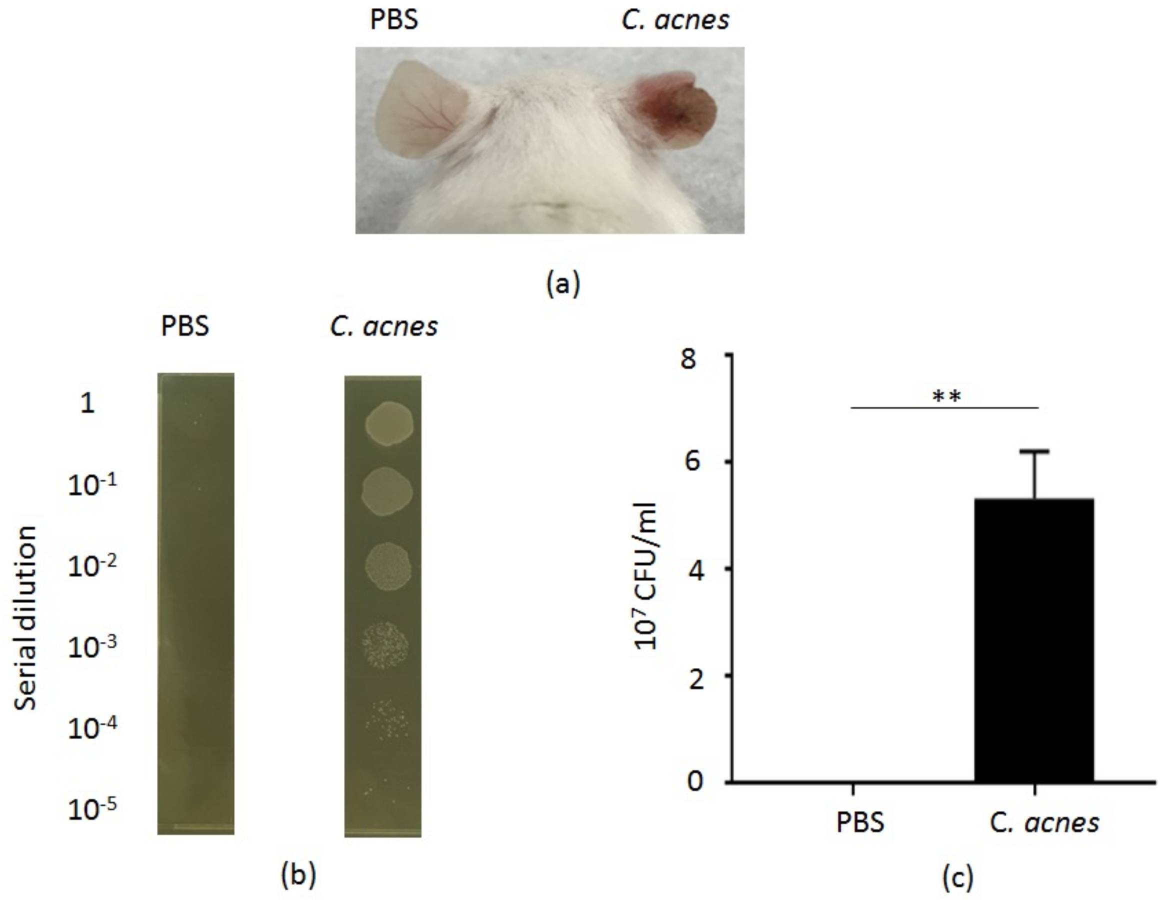

Figure S1. Detection of *C. acnes* isolated from mouse ears. (a) The ears of ICR mice were injected intradermally with *C. acnes* (ATCC 6919) (10^7 CFU/10μl) (right ear) or 10μl PBS (left ear) for 3 days. (b) The. (b, c) CFUs of *C. acnes* in PBS- or *C. acnes*-injected ears were quantified by serial dilutions (1:10–1:10^5) of homogenates on RCM plates. * *p < 0.001. (two-tailed t-tests).

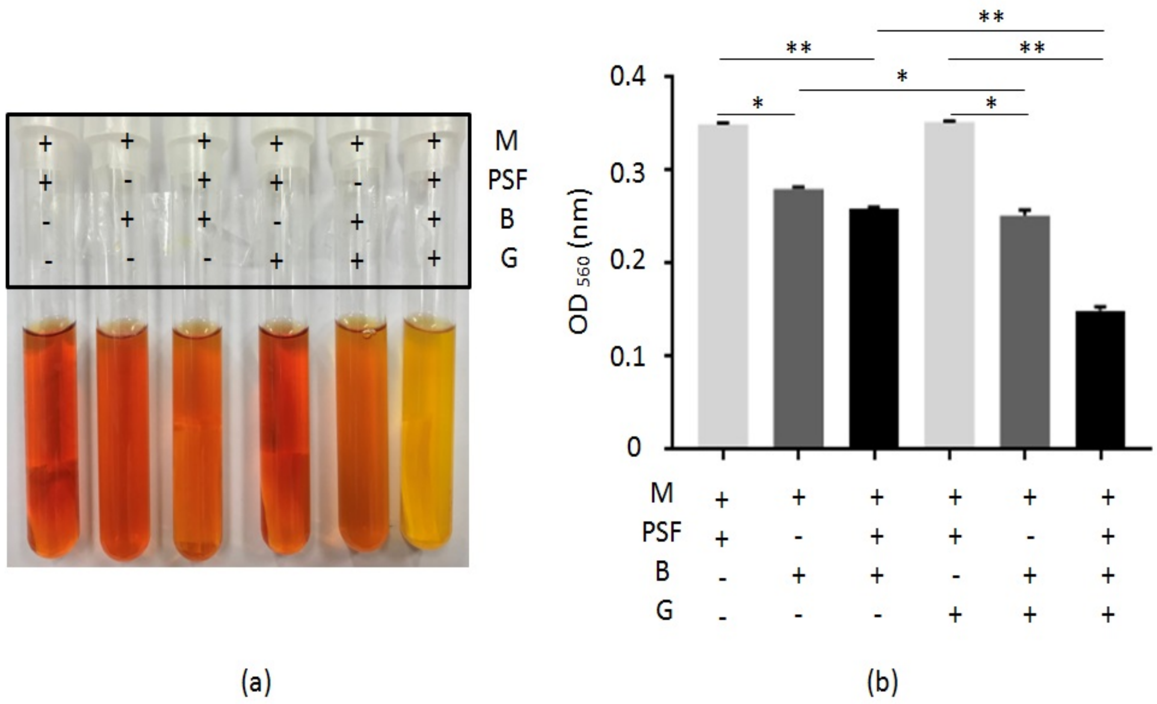

Figure S2. Fermentation activity of *S. epidermidis* in the presence or absence of glycerol. (a) PSF MTAM loaded-bacteria (B) (10^5 CFU/ml) were incubated in phenol red-containing rich media (M) in the presence or absence of glycerol (G). Rich media with PSF MTAM (PSF) alone or bacteria alone were included as controls. A yellow color change in media is indicative of extensive fermentation of bacteria. (b) The OD_{560} values in the rich media 3 days after bacterial culture were quantified. Results were illustrated as the mean ± SD of three independent experiments. *P<0.01; **P<0.001 (two-tailed t-tests).

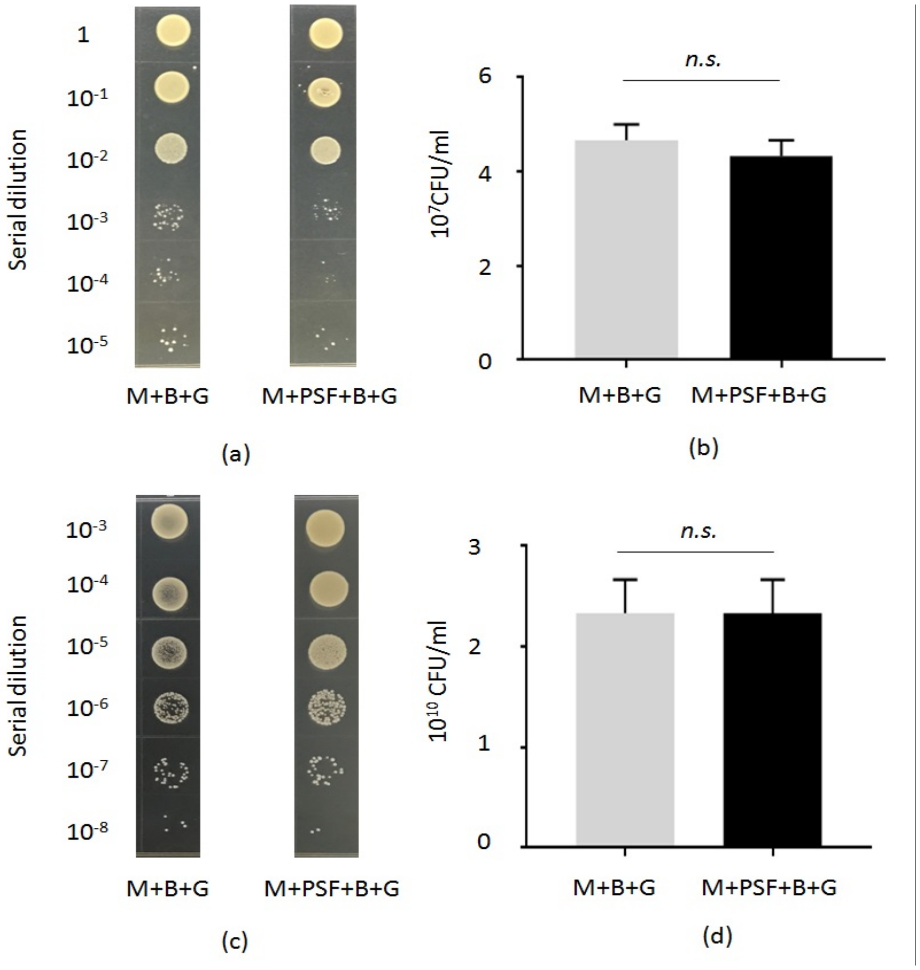

Figure S3. The growth of *S. epidermidis* suspended in media or encapsulated in PSF MTAM (PSF). TSB media (M) with 2% glycerol (G) were used for growth of suspended *S. epidermidis* (10^5 CFU/ml) (M+B+G) or PSF MTAM encapsulated *S. epidermidis* (M+PSF+B+G) for 10 (a, b) or 20 (c, d) h. The PSF MTAM encapsulated by *S. epidermidis* was homogenized before bacterial counts. The CFUs were enumerated by plating serial dilutions of culture media or PSF MTAM on agar plates. n.s. = non significant. (two-tailed t-tests).

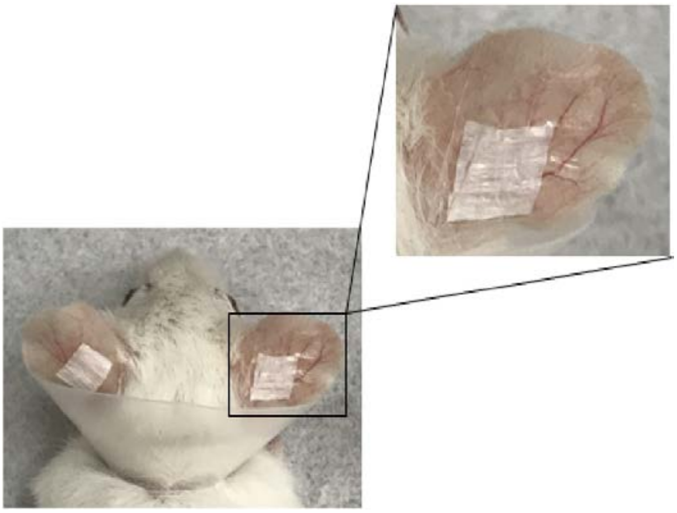

Figure S4. The *C. acnes*-injected ears of ICR mice were topically applied with PSF MTAM encapsulated with (right ear; zooming in within a panel) or without (left ear) *S. epidermidis.* An Elizabethan collar used to prevent mice from scratching out the PSF MTAM.
